# Characterization of the Impacts of Living at High Altitude in Taif: Oxidative Stress Biomarker Alterations and Immunohistochemical Changes

**DOI:** 10.3390/cimb44040110

**Published:** 2022-04-09

**Authors:** Mohamed Mohamed Soliman, Adil Aldhahrani, Fayez Althobaiti, Mohamed Mohamed Ahmed, Samy Sayed, Saqer Alotaibi, Mustafa Shukry, Ahmed M. El-Shehawi

**Affiliations:** 1 Clinical Laboratory Sciences Department, Turabah University College, Taif University, Taif 21995, Saudi Arabia; a.ahdhahrani@u.edu.sa; 2 High Altitude Research Center, Taif University, P.O. Box 11099, Taif 21944, Saudi Arabia; faiz@tu.edu.sa (F.A.); s.sayed@tu.edu.sa (S.S.); saqer@tu.edu.sa (S.A.); a.elshehawi@tu.edu.sa (A.M.E.-S.); 3 Department of Biotechnology, College of Science, Taif University, P.O. Box 11099, Taif 21944, Saudi Arabia; 4 Faculty of Veterinary Medicine, University of Sadat City, Sadat City 32958, Egypt; m_m_ahmed2000@yahoo.com; 5 Department of Science and Technology, University College-Ranyah, Taif University, P.O. Box 11099, Taif 21944, Saudi Arabia; 6 Department of Physiology, Faculty of Veterinary Medicine, Kafrelsheikh University, Kafrelsheikh 33516, Egypt; mostafa.ataa@vet.kfs.edu.eg

**Keywords:** high altitude, normal sea level, oxidative stress, hepatorenal dysfunction, gene expression, Taif

## Abstract

At high elevations, the human body experiences a number of pathological, physiological, and biochemical changes, all of which have adverse impacts on human health and organ vitality. This study aimed to investigate the alterations in the liver and kidney biomarkers, oxidative stress markers, gene expression, and cellular histology of rats maintained at high altitudes and normal sea level. A total of twenty male Wistar rats at 2 months of age were randomly assigned to two groups. The rats in group A were maintained at normal sea level in Jeddah, whereas rats in group B were maintained in an area in Taif 2600 m above sea level. After 2 months of housing, orbital blood samples were collected for the analysis of significant biochemical indicators of oxidative stress biomarkers of the liver and kidneys. Liver and kidney tissues from both groups were taken to examine the hepatorenal changes occurring at the biochemical, histological, immunohistochemical, and genetic levels. The results revealed substantial increases in the serum levels of liver and kidney biomarkers (GPT, GOT, urea, and creatinine) and decreases in the serum levels of antioxidant biomarkers (*SOD*, *catalase*, *GSH*, and *NO*). In parallel, the levels of the malondialdehyde (*MDA*) tissue damage marker and inflammatory cytokines (*IL-1β*, *TNF-α*, and *IFN-γ*) were increased in the high-altitude group compared to the normal sea level group. In addition, there were significant alterations in the oxidative and inflammatory status of rats that lived at high altitude, with considerable upregulation in the expression of hepatic *VEGF*, *type 1 collagen*, *Cox-2*, *TNF-α*, and *iNOS* as well as renal *EPASI*, *CMYC*, *HIF-α*, and *EGLN-2* genes in the high-altitude group compared with controls housed at normal sea level. In conclusion, living at high altitude induces hepatorenal damage and biochemical and molecular alterations, all of which may serve as critical factors that must be taken into account for organisms living at high altitudes.

## 1. Introduction

Hypoxia is defined as a state in which an organism, its organs, or its cells cannot readily obtain the oxygen they need. Hypoxia causes different negative impacts on organs, including physiological, biochemical, hematological, and molecular changes in a way to adapt to the extreme environmental conditions [1]. A variety of mechanisms must be activated to ensure the subject’s energy level remains stable under these condition. Hypoxia is associated with a state of high blood pressure, which helps in improving oxygen distribution across red blood cells [2]. Heart function suffers as a result of oxidative stress caused by intermittent hypercapnic hypoxia [3]. When an organism is exposed to hypoxic circumstances, systemic and cellular responses are activated in an attempt to meet oxygen demands [4].

Hypoxia has a significant impact on people’s health and daily activities at high altitudes because it profoundly affects physiology and causes pathological alterations in the body [5]. Drug absorption and distribution are closely linked to these changes induced by hypoxia [6]. As a result, pharmacokinetics cannot function without arterial blood gas analysis. Blood rheology, distribution, medication metabolism, and excretion are all affected [7]. As evidenced by growing studies, hypoxia appears to trigger an inflammatory response [8]. More than 140 million individuals around the world live at high altitudes [1], and living at high altitude has the potential to induce organ dysfunction syndromes that are caused by hypoxia. Organ dysfunction syndromes can cause severe intestinal barrier dysfunction, encourage bacterial and endotoxic translocation, and trigger an inflammatory response throughout the body [9]. Short- or long-term exposure at high altitudes induces respiratory, cardiovascular, and endocrine system dysfunction [10]. The deiodination pathway, which regulates thyroid hormone levels in the blood, can be impacted by hypoxia [11].

The environmental characteristics at high altitude include, but are not limited to, decreases in ambient oxygen tension, increase in solar radiation, extreme diurnal ranges in temperature, arid climate, and poor soil quality, in which hypoxia is a significant factor affecting the activity of human life and organs vitality [12]. The effects of hypoxia on both animals’ and humans’ biochemical, physiological, and metabolic systems is a topic of interest for further investigation through research [13].

Elevations above sea level are classified as high altitude (2000 to 4000 m), very high altitude (4000 to 5500 m), and extremely high altitude (>5500 m). These altitudes affect normal physiology and health due to the low partial pressure of oxygen [14]. Hypoxia develops due to this low oxygen pressure [15]; therefore, the body adapts through several physiological and molecular strategies. The blood pressure is increased to provide more oxygen to the tissues [16]. On the other hand, rapid changes in blood flow cause fluid leakage from the capillaries, leading to the development of serious conditions such as high-altitude pulmonary edema and high-altitude cerebral edema [17]. Cells can sense hypoxia via oxygen sensor prolyl hydroxylases [16], which activate the transcription factor hypoxia-inducible factor 1 (HIF-1) [18]. HIF-1 can relay the hypoxia signal and trigger adaptive hypoxic responses throughout the body [16,19].

Taif is located about 1700–2500 m above sea level and hosts resorts for national and international tourists. The impact of high altitude on global gene expression and the levels of biochemical and oxidative stress biomarkers must be characterized to provide residents with relevant information about the effects of hypoxia on their health. Therefore, the present study aimed to examine and compare the impacts of living at a high altitude (Taif) and at sea levels (Jeddah) on Wistar rats in terms of oxidative stress and inflammatory, antioxidant, and hepatorenal biomarkers. The changes in both the histology and immunohistochemistry of genes associated with hypoxia and oxidative stress in the liver and kidney were compared in groups housed at either high or normal sea levels.

## 2. Materials and Methods

### 2.1. Animal Handling and Experimental Design

Twenty albino rats were kept at room temperature with free access to food and water at the Biotechnology Department, Taif (High altitude), and King Fahad Institute of Research in Jeddah (sea level) following instructions from Taif University’s Institutional Animal Care and Use Committee, who authorized all animal use. Rats were birthed at normal sea levels at Jeddah. Rats were maintained for 2 months and weighed 150–170 g. Next rats were divided into two groups: group A (normal sea level group) was kept at Jeddah (sea level) for an additional 2 months; group B (high-altitude group) was taken to Taif (high altitude) and maintained for an extra 2 months. At the end of experimental study (2 months), rats were euthanized by decapitation following anesthetization. A non-heparinized Vacutainer tube was used to collect orbital blood, which was then centrifuged at 3000× *g* for 10 min. Samples were stored at −20 °C before being analyzed for blood chemistry and metabolite changes. Sliced and cleaned liver and kidney samples were then rinsed in cold saline to remove any remaining debris. For histological and immunohistochemical examination, tissue samples were stored in a 10% neutral buffer formalin solution or QIAzol for RNA extraction and real-time PCR.

### 2.2. Serum Biochemical Parameters

Superoxide dismutase (*SOD*), glutathione (*GSH*), catalase, and malondialdehyde (*MDA*) were measured using a colorimetric spectrophotometer based on the instructions provided by the manufacturers from Biodiagnostic Co. (Dokki, Giza, Egypt). Nitric oxide (NO) quantities were measured following the method of [20]. The activity of the glutamate pyruvate transaminase (GOT), glutamate oxaloacetate transaminase (GOT), and gamma-glutamyl transferase (GGT) enzymes in serum were tested using the corresponding kits from Spectrum Diagnostics, Obour City, Egypt (http://www.spectrum-diagnostics.com/new/p_01_02_Enzymes.php, accessed on 4 March 2022) according to the manufacturer’s instructions [21,22]. Urea levels were evaluated as described earlier [23]. Serum creatinine was assessed using modified Jaffe’s reaction as described earlier [24]. Uric acid was evaluated using Trinder’s enzymatic reaction as described in this study [25]. The Rat IL-6 ELISA Kits (ab100772) were obtained from Abcam Co. (Tokyo, Japan). The serum interferon-γ (*IFN-γ*) and tumor necrosis factor (*TNF-α*) levels were analyzed using specific ELISA kits (MyBioSource, San Diego, CA 92195-3308, USA) according to the manufacturer’s instructions.

### 2.3. Molecular Analysis Using qRT-PCR

Total RNA was extracted from liver and kidney tissues of groups A and B using an RNeasy Mini Kit (Cat# 74104, Hilden, Germany). RNA purity was measured using the A260/A280 ratio. RNA samples with A260/A280 ratios between 1.8 and 2 indicated high purity and were used in cDNA synthesis. The complementary DNA (cDNA) was synthesized with the HiSenScript kit by mixing 10 µL of 2 RT reaction solution, 1 µL of the enzyme, and 1 µg of total RNA and made up to final volume of 20 µL with RNase-free water. The reverse transcription reaction was made by incubating the mixture at 50 °C for 30 min and then at 85 °C for 10 min to inactivate the enzyme. The qRT-PCR was carried out on the PCR thermal cycler machine (iQ5 Real-time PCR, Bio-Rad, Hercules City, CA, USA) using a Quanti Fast SYBR Green PCR kit. The information for the primers, sequence, and product size is listed in Table 1. Each PCR reaction consisted of 1 μL cDNA and 10μL SYBR Green PCR Master Mix (Quanti Tect SYBR Green PCR Kit, Qiagen, Valencia, CA, USA), along with 1 μM of forward and reverse primer for each examined gene and nuclease-free H_2_O to a final volume of 20 μL. Reactions were run and analyzed in a Bio-Rad iQ5 real-time PCR machine. Real-time PCR conditions were: first denaturation at 95 °C for 10 min, followed by 40 cycles at 95 °C for 15 s (second denaturation), then annealing as shown in Table 1 for 60 stages. The critical threshold (Ct) of the target gene was normalized with quantities (Ct) of the housekeeping gene (β-actin) and GAPDH using the formula x = ^2 − ΔΔ^Ct, where there x = the fold difference.

### 2.4. Histopathological Evaluation of Hepatic and Renal Tissues

All rats were decapitated and necropsied at the end of the experiment [26,27], and representative hepatic and renal tissue was taken from each rat then incubated in 10% neutral buffered formalin for 24 h for preservation. After fixation, dehydration in ethanol, removal of impurities, impregnation, and embedding in paraffin wax were all carried out on the specimens before they were finally stained with hematoxylin and eosin and sectioned at a thickness of 4 µm [28]. The stained sections were evaluated microscopically. A multiparametric quantitative lesion assessments were separately performed on 40 images (8 images/5 rats per each group), as shown in last table.

### 2.5. Immunohistochemical Investigation of Heme Oxygenase-1 (HO-1) and Nuclear Factor Erythroid Factor 2-Related Factor-2 (Nrf-2)

For immunohistochemical analysis, slices were embedded in paraffin then deparaffinized and rehydrated. They were then soaked for 15 min in 2% H_2_O_2_ then washed in PBS to inhibit peroxidase activity. Non-specific binding sites were blocked using 5% bovine serum albumin. *HO-1* antibody (ab13243) and *Nrf-2* (ab31163) antibody were obtained from Abcam Corporation, Cambridge, MA, USA. These antibodies were diluted to 1:500 and added to the prepared slices from liver and kidney tissues. Sample slides were incubated overnight at 4 °C. The slides were then washed three times with PBS and incubated with a 1:2000 dilution of biotin-conjugated secondary antibody. These were developed using 3,3-diaminobezidine tetrahydrochloride and counterstained with hematoxylin [29]. In five non-overlapping, randomly selected microscopic fields, eight different snapshots were taken (8 shots per field for 5 different fields) for each gene to examine the immunoreactivity between groups A (sea level) and B (high altitude). These images were examined to measure the degree of immunoreactivity for the changes in the expression of *Nrf-2* and *HO-1* in the liver and kidneys, respectively [30]. The collective results for the immunoreactivity of the examined genes can be seen in last table of this study.

### 2.6. Statistical Analysis

The current data were analyzed using SPSS software for Windows, including a one-way ANOVA and Dunnett’s post hoc descriptive test (SPSS, IBM, Chicago, IL, USA). The data are presented as means with standard error (SEM). Here, *p* < 0.05 is considered to indicate statistically significant differences between the examined groups.

## 3. Results

### 3.1. Effects of High Altitude on Hepatorenal Function in Rats

The results in Table 2 reveal significant increases in the levels of liver biomarkers. There were substantial elevations in the GPT, GOT, and GGT in the high-altitude group compared with the normal sea level group. In addition, there were significant increases in the levels of the kidney injury markers creatinine, urea, and uric acid compared to the normal sea level group.

### 3.2. Effects of High Altitude on Serum MDA, Catalase, SOD, NO, and GSH Levels

Table 3 indicates significant increases in the MDA levels with prominent and significant decreases in the serum SOD, catalase, GSH, and NO levels in the high-altitude group relative to the normal sea level group.

### 3.3. Effects of High Altitude on Inflammatory Cytokine Biomarkers

The data presented in Table 4 show significant increases in the serum levels of *IL-6*, *TNF-α*, and *IFN-γ* for high-altitude rats compared to the normal sea level group. This confirms that high altitude (HA) is a stress factor that mediates inflammation and bodily responses in the subjects living in the Taif region.

### 3.4. Effects of High Altitude on Hepatorenal Gene Expression

**Figure 1:**
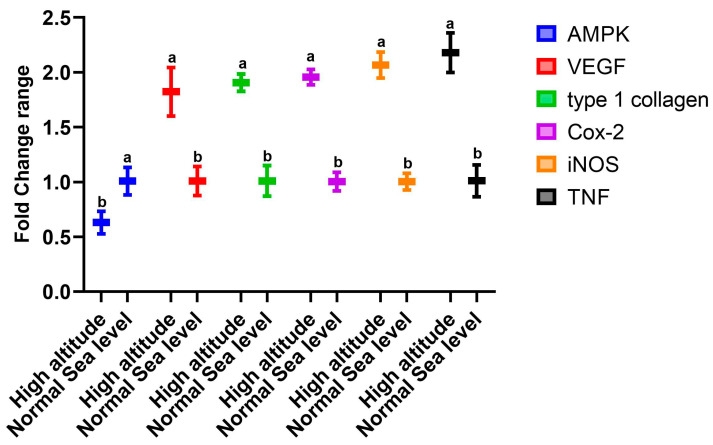
Impacts of high altitude on the expression levels of hepatic oxidative stress marker genes associated with hypoxia in the liver as assessed using quantitative real-time PCR. Bars indicate densitometric analysis of the expression levels of the examined genes for 10 different rats per group. Bars with different letters indicate significant differences between groups A (normal sea level) and B (high altitude) at *p* < 0.05.

**Figure 2:**
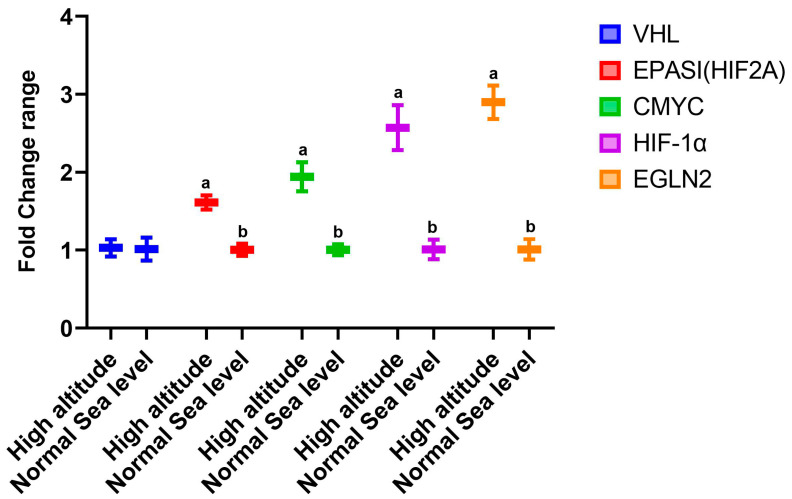
Impacts of high altitude on the expression levels of renal oxidative stress marker genes associated with hypoxia in the kidneys as assessed using quantitative real-time PCR. Bars indicate densitometric analysis of the expression levels of the examined genes for 10 different rats per group. Bars with different letters indicate significant differences between groups A (normal sea level) and B (high altitude) at *p* < 0.05.

The data presented in Figure 1 show the changes in the expression of genes in the livers of rats that lived at sea level (group A) and at high altitude (group B). There was significant upregulation in the mRNA expression levels of hepatic *VEGF*, type 1 collagen, *Cox-2*, *TNF-α*, and *iNOS* in group B compared to group A. However, there was downregulation in the expression of AMPK (Figure 1). Regarding the effect of high altitude on the expression of renal genes, the data in Figure 2 show that there was considerable upregulation in the expression of renal *EPASI*, *CMYC*, *HIF-α*, and *EGLN-2* in the high-altitude group (group B) compared with the normal sea level group (group A). There was no change in *VHL* mRNA expression between the groups.

### 3.5. Effects of High Altitude on Hepatorenal Histological Architect

**Figure 3:**
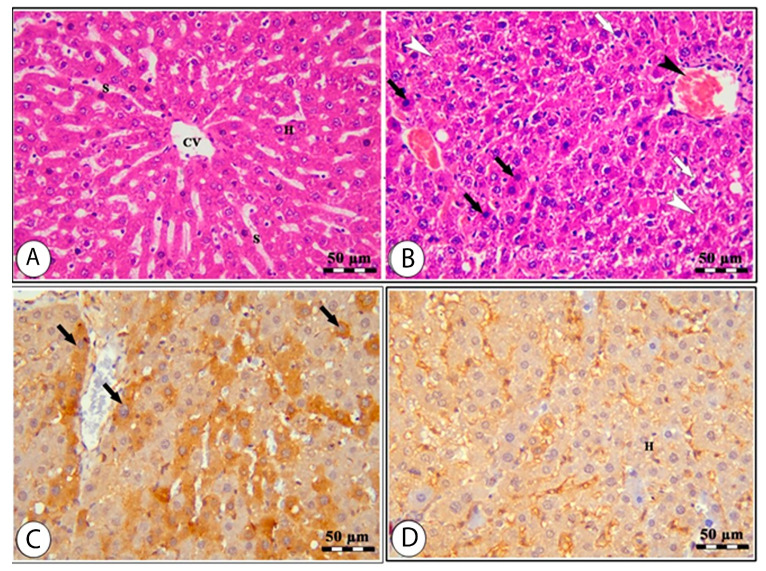
Liver sections of normal sea level (**A**,**C**) and high altitude (**B**,**D**) groups stained by H&E (**A**,**B**) and Nrf-2 IHC (**C**,**D**). The H&E-stained liver sample from the normal sea level group (**A**) shows normal hepatocytes (H), hepatic sinusoids (S), and the central vein (CV). The H&E-stained liver sample from the high-altitude group (**B**) shows a congested central vein (black arrowhead), areas of necrosis (white arrowheads), vacuolar degeneration (white arrows), pyknotic nuclei, and swelling of hepatocytes (black arrows). The *Nrf-2* IHC-stained liver sample from the normal sea level group (**C**) shows hepatocytes with *Nrf-2* positive staining (H). The *Nrf-2* IHC-stained liver sample from the high-altitude group (**D**) shows hepatocytes with less *Nrf-2* staining (black arrows).

**Figure 4:**
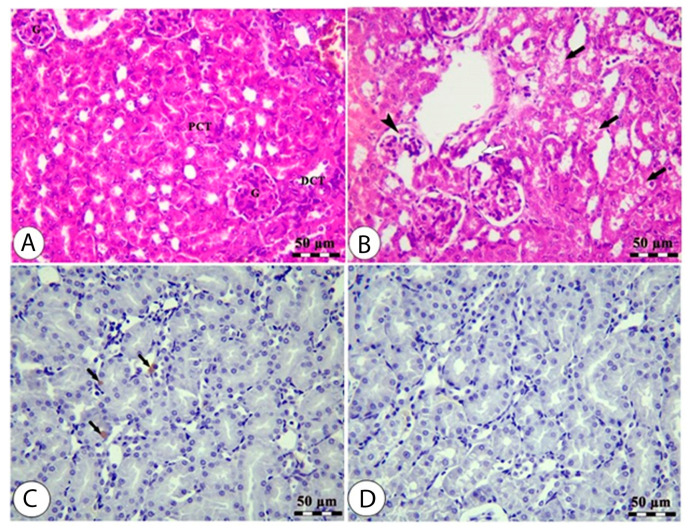
Kidney sections of normal sea level (**A**,**C**) and high altitude (**B**,**D**) groups stained by H&E (A&B) and *HO-1* IHC (**C**,**D**). The H&E-stained kidney sample from the normal sea level group (**A**) shows normal glomeruli (G), normal proximal (PCT), and distal (DCT) convoluted tubules. The H&E-stained kidney sample from the high-altitude group (**B**) shows degenerated glomeruli (black arrowhead), degenerated renal tubules (black arrows), and interstitial edema (white arrow). The *HO-1* IHC-stained kidney sample from the normal sea level group (**C**) shows renal tubules with mildly positive *HO-1* staining (black arrows). The *HO-1* IHC-stained kidney sample from the high-altitude group (**D**) shows renal tubules with less *HO-1* staining.

The histology of the hepatic tissue of the control group (Figure 3A) shows the presence of intact polyhedral hepatocytes arranged in a cord-like pattern radiating from the central vein, where each cord is separated from the others by hepatic sinusoids. The hepatic tissue samples of rats from the high-altitude group showed marked congestion of the central vein, nuclear pyknosis, swelling, vacuolar degeneration, and areas of hepatocyte necrosis (Figure 3B). The *Nrf-2* immunostaining expression was markedly decreased in the high-altitude group compared with the control group (Figure 3C,D), confirming the occurrence of hepatic oxidative stress. The renal cortex of the sea level group shows intact glomeruli with narrow capsular space in addition to intact proximal and distal convoluted tubules (Figure 4A). The kidney samples of the high-altitude group show marked degenerative changes in renal glomeruli and renal tubules (Figure 4B). *HO-1* immunostaining was markedly decreased in the high-altitude group compared with the control group, showing mild positive *HO-1* expression (Figure 4C,D). The immunoreactivity and lesion scoring results for both liver and kidney samples are presented in Table 5.

## 4. Discussion

The current study confirmed the feedback of living at high altitude in Taif compared to normal sea level at Jeddah for 2 months. We confirmed the changes in liver and kidney biomarkers at biochemical, cellular, and molecular levels. There were increases in the levels of oxidative stress markers for rats living at high altitudes compared to the normal sea level control group and decreases in *Nrf2* and *HO-1* immunohistochemistry in rats who lived at high altitude. The expression levels of hepatic *Nrf-2*, *HO-1*, *VEGF*, type 1 collagen, *Cox-2*, *TNF-α*, and *iNOS* together with renal *EPASI*, *CMYC*, *HIF-α*, and *EGLN-2* all showed upregulation. There were more changes in liver and kidney morphology in the high-altitude group than in the normal sea level group. In parallel, living in Taif induced a state of inflammation as reported by the increases in *IL-1β*, *TNF-α*, and *IFN-γ* levels at high altitudes compared to normal sea level.

Although the percentage of oxygen in inspired air remains constant at different altitudes, the partial pressure of inspired oxygen and the driving pressure for gas exchange in the lungs drops with the fall in atmospheric pressure at higher altitudes [31]. Living at high altitudes causes oxidative damage mediated by free radicals, which is a significant factor in the development of certain diseases and induction of undesirable metabolic reactions [32]. Proteins are inactivated by free radical reactions resulting from oxidative stress caused by high altitude living and carbonyls are formed. Carbonyls may cause cell and organ malfunction, particularly in the liver, due to functional impairment and cell death [33]. An organism’s physiological system undergoes a series of structural and functional alterations resulting from hypoxia. As a result, several pathophysiological diseases have been linked to HA-induced hypobaric hypoxia. The liver becomes more prone to oxidative stress when oxygen delivery is reduced due to hypoxia caused by high altitude [34]. Physiologically, this impairs the liver’s ability to detoxify drugs and impairs the functioning of liver cells, thereby explaining the reported alterations in liver activity in the group living in Taif.

Our data reveal that there were significant increases in the liver injury markers GPT, GOT, and GGT in the high-altitude group compared to the normal sea level group, and these results are in agreement with another study [35] in which increased levels of GPT, GOT, and lactate dehydrogenase (LDH) were observed in hypoxic rats, which was attributed to the disruption of lysosomes, an effect of hypoxia that leads to alterations in the permeability of cell membranes and subsequent enzyme release. Alterations in liver function considerably impact enzyme production [36] and activity, since the liver is a primary organ for drug metabolism [37]. In addition, another [36] report on the biochemical study of BUN, uric acid, and creatinine levels showed their concentrations were substantially increased at high altitudes. In that study, hypobaric hypoxia was found to reduce the glomerular filtration rate and renal blood flow at the same time, as it altered cell membranes and impacted the excretion of furosemide from the kidneys. Kidney cells were more vulnerable to oxidative stress and damage when the O2 concentration gradient was lower at high altitudes. The renal histology of rats confirmed this biochemical conclusion. These observations support our findings concerning the kidney injury markers creatinine, urea, and uric acid related to normal sea levels. High-altitude hypoxia damages renal cells by altering energy metabolism and membrane transport system performance, releasing free radicals, catalyzing enzymes, and damaging the cytoskeleton [38].

Data in the present study revealed that there were significant decreases in the *SOD* levels in the high-altitude compared to the normal sea level group; this result was consistent with [39], in which a decrease in the SOD enzymatic activity was reported to be due to decrease in oxidative phosphorylation of O2 in the mitochondrial respiratory chain, due to its limited availability at high altitudes [40].

Rats at high altitudes exhibited more significant levels of thiobarbituric acid reactive substances (*TBARS*). They had lower activities of *SOD* and *CAT* and lower levels of *GSH* than rats at low altitudes. Still, lower levels of *TBARS*, *SOD*, and *CAT* activities and higher *GSH* levels have been reported in kidney homogenates [41], findings that are in agreement with our results concerning the oxidative injury induced by hypoxic conditions. These findings show that the livers of animals living at high altitudes have higher levels of lipid peroxidation, and as a result more free radicals. Hypoxia has been shown to induce the expression of the steroidogenic acute regulatory protein and increase glucocorticoid release [42]. In the brains of rats treated with glucocorticoids, researchers detected decreased activity of antioxidant enzymes (systemic *SOD* and *CAT*) [43]. This could account for the drop in tissue levels of *SOD* and *CAT* that we observed in our research.

Hypobaric hypoxia is a symptom of high altitude considered an acute physiological stressor and a source of oxidative stress and oxidative damage caused by reactive oxygen species (ROS) in cells, tissues, or organs. ROS initiate lipid peroxidation, a cyclic event that generates free radicals by oxidizing polyunsaturated fatty acids in membranes [44], while *SOD*, *GSR*, and *GPX* also appear to be affected by hypoxia, which is known to impair enzyme activity [45]

Concerning the *NO* data, our results reveal that rats at high altitude showed decreased NO levels compared with those at normal sea levels, a result consistent with [46], where the authors reported that chronic hypoxia reduces *NO* concentrations in the serum of rats due to decreased *NO* bioavailability in the systemic circulation, which is a major contributor to hypoxia-induced endothelial dysfunction [47].

Hypoxia is associated with inflammation [44]. Inflammatory cytokines can activate nuclear factor kappa-B (*NFκB*), leading to increased inflammation and interleukin generation, suppressing the elevated pro-inflammatory markers (*NF-κB*, *TNFα*, and *IL6*) that might be responsible for adaptogenic potential during exposure to hypoxia [48]. In addition, cytokines such as *IL-1*, *IL-2*, *IL-4*, *IL-5*, *IL-6*, *IFN-γ*, and *TNF-α* are secreted in response to hypoxia [49]. These findings explain our obtained results concerning the cytokines in which there were significant increases in the serum *IL-6*, *TNF-α*, and *IFN-γ* levels of high-altitude rats compared to those in the normal sea level group. There are numerous molecular events and pathways that are associated with living at high altitude; our findings show that there was significant upregulation of hepatic *VEGF*, type 1 collagen, *Cox-2*, *TNF-α*, and *iNOS* in the high-altitude group compared with the normal sea level group and significant downregulation in hepatic AMPK mRNA expression. In parallel, the high-altitude group showed significant upregulation of renal *EPASI*, *CMYC*, *HIF-α*, and *EGLN-2* levels compared with the normal sea level group, and there were no changes in the *VHL* expression between the examined groups. In the cirrhotic liver, angiogenesis is mostly dependent on *VEGF* [50]. The expression of *VEGF* and *VEGF* receptors is upregulated in the regenerating rat liver and plays a significant role in the proliferation of sinusoidal endothelial cells, making it clear why induced *VEGF* expression is a frequent response to liver injury [51]. Collagen 1 expression might be directly induced by hypoxia in the initial stages [52].

Our findings are in harmony with others [53] that concluded that hypoxia enhances VEGF expression and collagen I expression in hepatic cells and suggested that it may play a role in advancing chronic liver disorders through its involvement in angiogenesis and fibrogenesis. A rise in *HIF-1* protein levels is a direct result of hypoxia, indicating that in the presence of intermittent hypoxic circumstances, systemic adaptation occurs gradually at the cellular level. The adaptation of cells or tissues to hypoxic conditions is assumed to be responsible for this rise [54]. In addition, it was found that green tea extract significantly inhibits hypoxia- and *HIF-1α-*related protein accumulation and the corresponding expression of *VEGF* at both mRNA and protein levels in HeLa and *HepG2* cells. These findings suggest that the suppression of hypoxia-induced VEGF expression by green tea extract and EGCG is due, at least in part, to their inhibitory effects on *HIF-1α* transactivation of the *VEGF* gene in HeLa cells [55].

Consistent with this, in rats vulnerable to hypoxia, the level of HIF-1 expression is statistically higher [56], which is accordance with the statement in [57] that inflammatory disorders are more likely to occur in animals that suffer from hypoxia. The results of our molecular pathway analysis are consistent with those of other studies in which *HIF*-mediated gene expression and caspase-3 were induced in hypoxia [58,59]. When hypoxia is detected, the body responds by producing *HIF*, a protein crucial for adaptation [60]. In addition, the authors of [61] reported the increased expression of *VEGF* receptors in prolonged exposure to hypoxia. In cells lacking the *VHL* gene, HIF-1 suppresses *c-Myc* activity, decreasing mitochondrial biogenesis and O2 consumption [62].

High-altitude exposure also stimulates the expression of enzymes that regulate fatty acid production. High-altitude exposure could significantly increase the expression of hepatic acetyl-CoA carboxylase-1 (*ACC-1*), a rate-limiting enzyme in fatty acid biosynthesis [63], with a significant reduction in the expression of *AMPK*, an enzyme reported to inhibit lipogenesis through suppressing the activity of *ACC-1* and malonyl CoA (*M-CoA*) [64], which supports our finding concerning hepatic *AMPK* mRNA expression. Renal inflammation and interstitial fibrosis are both exacerbated by intra-renal hypoxia. Renal tubular cells may release inflammatory cytokines or interact with macrophages, explaining the connection between *HIF-1* and renal interstitial inflammation [65]. Cell metabolism and proliferation are thought to be accelerated and induced by the transcription factor *c-Myc* [66]. Evidence suggests that several animal models of renal fibrosis show markedly elevated *c-Myc* protein expression [67]. The mRNA expression of *HIF-2A* increased in mice exposed to chronic and intermittent hypoxia [68].

Hypoxic conditions also increase HIF-1α and *VEGF* levels [69]. Studies have shown that *HIF-1* is an excellent indicator of hypoxia. Multiple genes are activated when hypoxia and other stimuli activate the *HIF-1* signaling cascade. Hypoxia-induced ROS production is a signaling chain involving *HIF-1* [70,71]. Free radicals are being produced due to chronic hypoxia [72], activating the transcriptional activity of *NFkB* in the nucleus by increasing its nuclear translocation. Pro-inflammatory cytokines, TNF, interleukins, *COX-2*, *iNOS*, as well as adhesion molecules such as vascular cell adhesion molecule-1, intercellular adhesion molecule-1, and E-selectin are produced when *NFkB* is activated [73]. Aside from endothelial cells, *EPAS1* expression has also been observed in the lungs, placenta, kidneys, heart, liver, small intestine, and other organs that regulate oxygen metabolism [74]. In genome-wide association studies, *EPAS1* was found to be linked to hypoxia adaption and decreased hemoglobin concentration in Tibetans [75]. The expression levels of *HIF-1* and *iNOS* are significantly linked. In a separate investigation, hypoxia boosted the expression of the *iNOS* gene in the pulmonary artery endothelial cells of rats through activating *HIF-1* [76]. 

Thus, *HIF-1* and iNOS may be necessary for the pathophysiology of hypoxia damage, and *HIF-1* controls iNOS expression [77]. Rats exposed to high altitude had higher *EGLN-1* and *PPAR* gene expression levels in their heart, liver, and kidneys. In addition, at high altitudes, the expression of the *HIF-2* protein in the liver, brain, and renal tissues was dramatically elevated. In the heart, liver, and kidneys, expression of prolyl hydroxylase domain-containing protein 2 (*PHD2*) and *PPAR* increased [78], which may be utilized as indicators of hypoxia. Hypobaric hypoxia significantly reduces *Nrf-2* expression. After being exposed to oxidative stress, the level of *HO-1* showed a comparable reaction consistent with that of *Nrf-2*. *Nrf-2* has been shown to function as a transcription factor that regulates cellular redox balance and promotes the production of antioxidant enzymes to protect cells from oxidative damage [79]. Under normal conditions, *Nrf-2* is sequestered in the cytoplasm by binding to the cysteine-rich Kelch-like *ECH*-associated protein 1 (*Keap1*), which is involved in the detoxification of reactive oxidants, the maintenance of cellular homeostasis, and the removal of a variety of exogenous and endogenous chemicals [80]. This confirms the downregulation in *Nrf-2* and *HO-1* immunoreactivity seen in liver and kidney immunohistochemistry.

## 5. Conclusions

**Figure 5:**
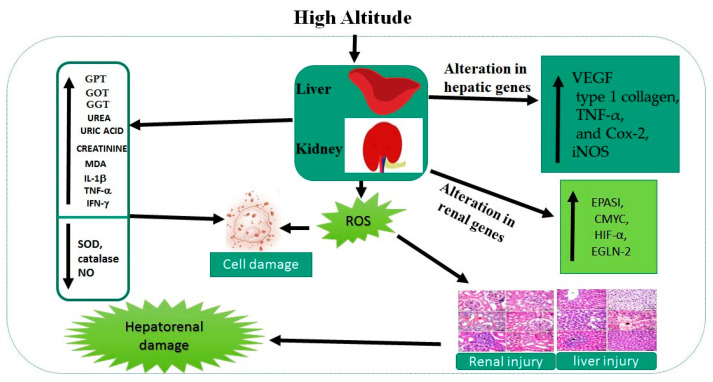
Collective impacts of living at high altitude on liver and kidney markers.

In summary, our results demonstrate that a hypoxia environment causes pathological changes in the liver and kidney that are evident from an increase in hepatic and renal injury markers and an increase in the levels of oxidative stress markers, together with the increased expression of cytokines and the activation of genes related to adaptation to hypoxia (namely hepatic *VEGF*, type 1 collagen, *Cox-2*, *TNF-α*, and *iNOS*) and renal *EPASI*, *CMYC*, *HIF-α*, and *EGLN-2* levels at high altitude. The altered biochemical and molecular markers are potentially significant therapeutic targets for the prevention or treatment of hepatorenal injury induced by living at high altitude. The collective impacts of living at high altitude are summarized in Figure 5.

## Figures and Tables

**Table 1:** Primer names, gene accession numbers, and sequences used for quantitative real-time PCR in rats.

Organ	Gene	Accession Number	Direction	Primer Sequence	Product Size (bp)	Annealing Temp (Tm °C)	Efficiency %	Slope
Liver	*iNOS*	NM_153629.1	Sense	TGGGTGAAAGCGGTGTTCTT	108	60	95.298%	−3.44
			Antisense	TAGCGCTTCCGACTTCCTTG				
	*TNF-α*	L19123.1	Sense	CAGCCGATTTGCCATTTCA	111	59	93.801%	−3.48
			Antisense	AGGGCTCTTGATGGCAGAGA				
	*Cox-2*	NM_017232.3	Sense	CTGAGGGGTTACCACTTCCA	209	61	98.435%	−3.36
			Antisense	TGAGCAAGTCCGTGTTCAAG				
	type 1 collagen	NM_021578.2	Sense	CAGTCGATTCACCTACAGCAC	198	58	92.349%	−3.52
			Antisense	GGGATGGAGGGAGTTTACACG				
	*VEGF*	AY033508.1	Sense	CAAACCTCACCAAAGCCAGC	118	60	96.064%	−3.42
			Antisense	TTCTCCGCTCTGAACAAGGC				
	*AMPK*	NM022627.1	Sense	TCTCGGGGTGGTTCGGTG	131	59	97.236%	−3.39
			Antisense	GGGGACAGGATTTTCGGATT				
	β-actin	NM 031144	Sense	AGGAGTACGATGAGTCCGGC	71	58	95.289%	−3.55
			Antisense	CGCAGCTCAGTAACAGTCCG				
	GAPDH	NM_017008.4	Sense	TCAAGAAGGTGGTGAAGCAG	123	58	95.2%	−3.44
			Antisense	AGGTGGAAGAATGGGAGTTG				
Kidney	*EGLN-2*	NM_001004083.1	Sense	TCAGTCCGTCCGTCTGGC	162	60	99.251%	−3.34
			Antisense	GCCTCGTGTGGGGCAG				
	*HIF-1α*	NM_024359.2	Sense	AAGCAGCAGGAATTGGAACG	178	59	99.664%	−3.33
			Antisense	TCATCCATTGACTGCCCCAG				
	*C-MYC*	NM_012603.2	Sense	ACTCGGTGCAGCCCTATTTC	187	60	98.435%	−3.36
			Antisense	GTAGCGACCGCAACATAGGA				
	*EPASI* (*HIF2A*)	NM_023090.2	Sense	GACTGTATGGTCATCTCAGCGG	193	59	97.632%	−3.38
			Antisense	TGCAAGACGCCAAAAGAGAG				
	VHLEL	NM_052801.2	Sense	CGGAACTGTTTGTGCCATCC	187	60	93.801%	−3.48
			Antisense	CGCACATTTGGGTGGTCTTC				
	β-actin	NM 031144	Sense	AGGAGTACGATGAGTCCGGC	71	58	94.289%	−3.55
			Antisense	CGCAGCTCAGTAACAGTCCG				
	GAPDH	NM_017008.4	Sense	TCAAGAAGGTGGTGAAGCAG	123	58	95.2%	−3.44
			Antisense	AGGTGGAAGAATGGGAGTTG				

Genes abbreviations used in this study were as follows: *iNOS*, inducible nitrous oxide; *TNF-α*, tumor necrosis factor alpha; *Cox-2*, cyclooxygenase-2; *VEGF*, vascular endothelial growth factor; *AMPK*, activated mitogen protein kinase; *EGLN-2*, Egl-9 family hypoxia-inducible factor 2; *HIF-1α*, hypoxia-inducible factor 1 alpha; *C-MYC*, c-myelocytomatosis; *EPASI*(*HIF2A*), hypoxia-inducible factor 2A; VHLEL, Von Hippel–Lindau tumor suppressor. GAPDH, glyceraldehyde-3-phosphate dehydrogenase.

**Table 2:** Alterations in serum liver and kidney biomarkers in groups living at normal sea levels and at high altitude.

	Normal Sea Level	High Altitude
*GPT* (U/L)	17.3 ± 0.3 ^a^	58.3 ± 1.5 ^b^
*GOT* (U/L)	20.4 ± 0.4 ^a^	51.6 ± 0.8 ^b^
*GGT* (U/L)	9.4 ± 0.3 ^a^	20.2 ± 1. 1 ^b^
*Creatinine* (mg/dL)	0.5 ± 0.02 ^a^	1.7 ± 0.2 ^b^
*Urea* (mg/dL)	18.4 ± 0.6 ^a^	43.1 ± 1.6 ^b^
*Uric acid* (g/dL)	4.7 ± 0.15 ^a^	13.7 ± 0.4 ^b^

Values are means ± SEM for 10 different rats per group. Values with different letters indicate significant differences between groups A (normal sea level) and B (high altitude) at *p* < 0.05.

**Table 3:** Alterations in serum MDA, catalase, SOD, NO, and GSH levels between groups living at normal sea level and at high altitude.

	Normal Sea Level	High Altitude
*MDA* (nmol/mL)	12.1 ± 0.3 ^a^	32.1 ± 1.4 ^b^
*SOD* (U/mL)	3.4 ± 0.05 ^a^	1.8 ± 0.01 ^b^
*Catalase* (U/L)	200.0 ± 9.0 ^a^	132.1 ± 4.9 ^b^
*NO* (ng/mL)	15.2 ± 1.7 ^a^	10.5 ± 0.7 ^b^
*GSH* (U/mL)	3.3 ± 0.2 ^a^	1.3 ± 0.1 ^b^

Values are means ± standard error (SEM) for 10 different rats per group. Values with different letters indicate significant differences between groups A (normal sea level) and B (high altitude) at *p* < 0.05.

**Table 4:** Alterations in serum IL-6, TNF-α, and IFN*γ* concentrations in groups living at normal sea level and at high altitude.

	Normal Sea Level	High Altitude
*IL-6* (pg/mL)	64.2 ± 0.3 ^a^	157.6 ± 4.2 ^b^
*TNF-α* (pg/mL)	515.2 ± 13.0 ^a^	775.5 ± 5.8 ^b^
*IFN-γ* (pg/mL)	661.6 ± 15.3 ^a^	842.2 ± 19.9 ^b^

Values are means ± SEM for 10 different rats per group. Values with different letters indicate significant differences between the group A (normal sea level) and B (high altitude) at *p* < 0.05.

**Table 5:** Histopathological changes as described by H&E and immunoexpression of *HO-1* and *Nrf-2* in the hepatic and renal tissues of rats who lived at sea and high altitude levels.

Organ	Lesion and Immunoexpression	Normal Sea Level	High Altitude
Liver	Congestions	0 ^a^	7.1 ± 0.9 ^b^
	Fatty change	0 ^a^	3.5 ± 0.6 ^b^
	Inflammatory infiltrate	0 ^a^	5.4 ± 0.3 ^b^
	Vacuolar and hydropic degeneration	0 ^a^	8.76 ± 1.4 ^b^
	Single-cell necrosis	0 ^a^	3.1 ± 0.9 ^b^
	Nrf-2 immunoreactivity	13.3 ± 0.5 ^a^	4.8 ± 0.8 ^b^
Kidney	Glomerular congestion	0 ^a^	7.1 ± 1.2 ^b^
	Interstitial congestion	0 ^a^	5.1 ± 0.7 ^b^
	Glomerular necrosis	0 ^a^	3.4 ± 0.7 ^b^
	Tubular attenuation	0 ^a^	9.7 ± 1.3
	Tubular vacuolation	0 ^a^	10.3 ± 2.5 ^b^
	Tubular necrosis	0 ^a^	5. 7 ± 0.4 ^b^
	Cast formation	0 ^a^	4.1 ± 0.9 ^b^
	Inflammatory cell infiltrate	0 ^a^	1.1 ± 0.2 ^b^
	HO-1 immunoreactivity	11 ± 0.5 ^a^	2.6 ± 0.8 ^b^

Values are means ± SE for 5 rats/group. Means within the same row (in each parameter) carrying different superscript letters are significantly different at *p* < 0.05.

## Data Availability

All data sets obtained and analyzed during the current study are available in the manuscript.

## Abbreviations

*ACC-1*: Acetyl-CoA carboxylase-1; *AMPK*: activated mitogen protein kinase; *CAT*: catalase; cDNA: complementary DNA; *C-MYC*: c-myelocytomatosis; *Cox-2*: cyclooxygenase-2; CT: cycle threshold; EGLN2: Egl-9 family hypoxia-inducible factor 2; *EPASI*(*HIF2A*): hypoxia-inducible factor 2A; GOT: glutamate oxaloacetate transaminase; GPT: glutamate pyruvate transaminase; GPX: glutathione peroxidase; GSR: glutathione reductase; HACE: high-altitude cerebral edema; HIF-1: hypoxia-inducible factor-1; *HIF-1**α*: hypoxia-inducible factor 1 alpha; *HO-1*: heme oxygenase; *iNOS*: inducible nitrous oxide; *Keap1*: Kelch-like ECH-associated protein 1; LDH: lactate dehydrogenase; MDA: malondialdehyde; M-CoA: malonyl CoA; NF-κB: nuclear factor kappa-B; *Nrf-2*: nuclear factor-erythroid factor 2-related factor 2; *PHD2*: prolyl hydroxylase domain-containing protein 2; *PPAR*: peroxisome proliferator-activated receptor; qRT-PCR: quantitative real-time PCR; ROS: reactive oxygen species; SOD: superoxide dismutase; VEGF: vascular endothelial growth factor; TBARS: thiobarbituric acid reactive substances; TNF-α: tumor necrosis factor alpha; VHLEL: Von Hippel–Lindau tumor suppressor.
